# Refugee-centered framework for design of digital health interventions: a qualitative study with Ukrainian refugee women in Norway

**DOI:** 10.3389/fdgth.2026.1886871

**Published:** 2026-07-27

**Authors:** Andrea Porras Elizo, Ashis Jalote-Parmar

**Affiliations:** Department of Design, Faculty of Architecture and Design, NTNU Norwegian University of Science and Technology, Trondheim, Norway

**Keywords:** digital health interventions, digital health navigation, health equity, refugee-centered framework, Ukrainian refugee women, user-centered design

## Abstract

**Introduction:**

Ukrainian refugee women face multiple barriers when accessing healthcare systems in host countries, including language challenges, differences in healthcare system structures, prior experiences, and issues of trust and expectations. Digital health interventions have been increasingly used to support access to health information and services for refugee populations. However, evidence remains limited regarding how refugee women understand, navigate, and engage with digital health within host country healthcare systems. This study aims to identify the needs and expectations of Ukrainian refugee women in relation to digital health and healthcare navigation in Norway, and to develop a refugee-centered, empirically grounded framework to inform the design of digital health interventions that support equitable healthcare access.

**Methods:**

A qualitative design was employed, including five age-segmented focus group discussions with thirty Ukrainian refugee women aged 18–24, 25–54, and 55+ years. Participants also completed a pre-study questionnaire documenting demographic characteristics and prior use of digital health. Data were analyzed thematically and subsequently interpreted through a user experience framework as an analytical lens.

**Results:**

Nine overarching themes were identified, describing navigation challenges shaped by prior system experiences, age-related differences in information use, prevention priorities, trust, language accessibility, the need for step-by-step guidance, information structure and usability, digital overload, and the cautious use of artificial intelligence and digital contact tools.

**Discussion:**

These findings were further synthesized into user-centered design requirements for digital health interventions. Based on these findings, a refugee-centered framework is proposed for designing digital health interventions. The framework identifies six core components: navigation and pathway guidance, age-responsive design, trust and verification, language and cultural adaptation, artificial intelligence-supported features, and cognitive ease, accessibility, and universal design.

**Conclusions:**

This study contributes to more equitable and user-centered digital health design by translating the lived experiences of Ukrainian refugee women into actionable design principles that support navigable, trustworthy, and linguistically accessible healthcare systems.

## 1 Introduction

The large-scale displacement of Ukrainian refugees has created sustained challenges for healthcare systems across Europe, particularly in supporting access to care for newly resettled populations (1). Europe, as the most affected continent, has received more than five million refugees, who have been dispersed across multiple countries (2). This ongoing displacement of Ukrainian refugees has contributed to the transition of healthcare systems from an emergency response to the long-term integration of this population into the system. However, this sudden massive arrival of refugees also placed significant pressure on healthcare systems, as illustrated by Germany, which received more than one million refugees in less than a year, resulting in increased healthcare strain (3). A similar situation occurred in Poland, which is among the European countries most affected by Ukrainian migration and had limited public expenditure on healthcare (4). As a result, many countries have experienced similar pressures on healthcare services, including workforce shortages, longer waiting times, and challenges in integrating refugees into existing care pathways (5).

The Nordic-Baltic region has hosted a large number of Ukrainian refugees since 2022, and has worked to ensure their access to health services, with some variation in eligibility and service provision between countries (6). For example, the Swedish Migration Studies Delegation has proposed coordinated policy measures to expand access to mental health support, and culturally adapted services for displaced Ukrainians (7). In the context of the present study, Norway is of particular interest, as it hosts more than 86,000 Ukrainians with valid collective protection as of 2026 (8). Migration patterns suggest continued arrivals, with recent findings indicating as reported by the Norwegian Institute for Urban and Regional Research, which found that 47% of Ukrainian refugees plan to stay in Norway, and one third are considering bringing more family members to the country (9). In this context, the healthcare system may need to adapt to a long-term scenario of sustained demand. At the same time, differences between healthcare systems create barriers to navigation, contributing to confusion and frustration among Ukrainian refugees. For example, their childbirth experiences in Ukraine influenced their expectations of maternal care in Norway and made the unfamiliar system hard to navigate (10). Difficulties accessing specialist healthcare services, long waiting times, communication barriers, and limited access to medications have been identified as common barriers to accessing the Norwegian healthcare system (11). Similar barriers have been reported across Europe, including waiting times, information gaps, and service costs (12).

As a consequence of these pressures, healthcare systems must balance increasing demand with the effective integration of Ukrainian refugees into existing healthcare services (13). The World Health Organization (WHO), the International Organization for Migration (IOM), and the European Union (EU) have joined efforts to support and strengthen healthcare systems overloaded by the arrival of Ukrainian refugees (14). While such initiatives have provided important short-term support, there remains a need for complementary and scalable solutions that address long-term access and navigation challenges. In this context, digital health interventions (DHIs) offer promising opportunities to support health promotion and improve access to healthcare information, as demonstrated in other refugee settings (15–29). These interventions include a range of solutions such as mobile health applications, telehealth services, online health information platforms, and digital screening or self-management tools designed to support access, navigation, and care coordination. In this paper, DHIs are defined as digital health platforms through which information is accessed, interpreted, and acted upon within healthcare systems.

DHIs have increasingly emerged as important tools for improving healthcare accessibility and communication among refugee populations. For millions of displaced individuals, access to healthcare in host countries progressively depends on digital health. Within this specific context, where ongoing forced migration is expected to continue, these healthcare systems should be equipped to adapt constantly to emerging needs (30). DHIs support this adaptation by providing a range of functionalities, including health reminders (15–18), educative videos (18, 19), digital storytelling (20), algorithms (21), virtual reality solutions (22), or new virtual health assistants (23). DHIs have been widely used to support refugee populations in areas such as vaccination (15, 18, 20–22), cancer screening (16, 19, 23, 24), and mental health (17, 21, 25–28). Digital transformation in healthcare is therefore gradually emphasized at the policy level, including by the EU, which promotes e-health services grounded in inclusiveness and solidarity (5). For Ukrainian refugees, culturally and linguistically appropriate DHIs may offer a scalable means of supporting healthcare navigation and access to preventive information.

However, despite a growing body of research, there remains limited evidence on how refugees themselves identify, interpret, and act upon digital health information within unfamiliar healthcare systems. Existing studies predominantly focus on the implementation or effectiveness of specific DHIs (29, 31), while considerably less attention has been paid to refugees' lived experiences of accessing health information, navigating unfamiliar healthcare structures, and negotiating trust, language, and system expectations through digital means. This gap highlights the need to understand digital health not only as a tool, but as a socially situated practice modeled by prior experiences, expectations, and contextual constraints. Ukrainian refugees originate from a healthcare context with relatively high levels of digital health integration (32, 33). Prior to displacement, healthcare access in Ukraine was increasingly mediated through digital health, supported by the rapid expansion of the national eHealth infrastructure and telemedicine services (34). This digitalisation included the use of online platforms such as Helsi, Doc.ua, and similar systems for booking appointments, accessing medical records, and obtaining electronic prescriptions. These digitally embedded practices shape expectations and influence how refugees engage with and navigate healthcare systems in host countries, often resulting in mismatches in system understanding and use. Additionally, women's perspectives are often underrepresented, despite their central role in managing personal, family, and children's health following forced displacement (35).

In host-country contexts such as Norway, where Ukrainian refugee women (URW) constitute the majority of the refugee population (36), there is a need to understand how digital health is accessed, interpreted, and translated into action, and how age-related differences influence these practices. Importantly, few studies move beyond needs identification to systematically translate user-derived insights into design-ready frameworks that can effectively guide the development of DHIs. Addressing this gap is essential for ensuring that future DHIs are not only available, but also navigationally supportive, trustworthy, gender responsive, and grounded in the lived realities of refugee women. This study aims to explore the needs and expectations of URW regarding digital health and healthcare navigation in Norway and to develop a refugee-centered, empirically grounded framework to inform the design of DHIs.

## 2 Methods

### 2.1 Qualitative approach and research paradigm

This article reports on the digital health and navigation component of broader research study examining URW's health-seeking behaviors and access to healthcare in Norway. The semi-structured discussion guide comprised five topic areas: first encounters with health services, health literacy, health-seeking behaviors, digital health, and barriers and facilitators to access. The present study focuses specifically on the digital health component, examining participants' experiences with DHIs in both Ukraine and Norway, as well as their preferences for digital health use and healthcare navigation. A companion paper examines complementary aspects of this dataset, including health-seeking trajectories, health system navigation and health literacy (37). This qualitative study adopts an interpretivist-constructivist paradigm, emphasizing the importance of participants' lived experiences, interactions and context in co-constructing an understanding of digital healthcare access.

### 2.2 Researcher characteristics and reflexivity

Focus groups discussions (FGDs) were moderated by a Ukrainian interpreter trained in qualitative research and experienced in facilitating FGDs. The Ukrainian transcription and verification team consisted of two native Ukrainian interpreters. The analysis team included two researchers trained in design research. No researcher or member of this study had a supervisory or clinical relationship with participants. Reflexive attention to researcher influence was maintained through analytic memos and team discussions, in alignment with the Standards for Reporting Qualitative Research (SRQR) (38).

### 2.3 Context

The study was conducted in Norway, within a municipality that has received a substantial number of Ukrainian refugees following the 2022 full-scale invasion of Ukraine. The setting provided an appropriate context due to its established municipal integration services, adult-education centers and community organizations that support newly arrived refugees. Participants were at different stages of settlement, enabling the exploration of experiences with both healthcare access and digital health engagement.

### 2.4 Sampling strategy

Participants were recruited voluntarily, using purposive and snowball sampling to capture a diverse group of URW. Recruitment materials were distributed across community settings commonly attended by URW, including adult education centers, cultural centers and community support organizations as well as through relevant social-media channels, and local Ukrainian community groups. Two Ukrainian interpreters supporting the study shared the study announcement within local Ukrainian networks and online community groups to broaden outreach. Study information and the digital recruitment questionnaire were provided in Ukrainian to ensure accessibility. This sampling approach enabled the inclusion of URW with variation in age, migration histories, and healthcare experiences, and was appropriate for identifying individuals actively navigating the Norwegian healthcare system.

### 2.5 Ethical issues pertaining to human subjects

The study was conducted in accordance with the Declaration of Helsinki, the Norwegian Personal Data Act/GDPR and guidance from the Norwegian Agency for Shared Services in Education and Research Sikt (formerly NSD). Ethics approval was granted by Sikt/NSD, registration number 669383. It was also submitted to the Regional Committee for Medical and Health Research Ethics (REK), which confirmed that formal approval was not required because no directly identifiable personal health data were collected (reference 954842). This study did not constitute a clinical trial. The study followed the highest ethical standards: participation was voluntary and all participants provided written informed consent prior to participation. Principles of informed consent were strictly followed. Participants were fully informed in their native language about the study's purpose, procedures, potential risks and benefits, confidentiality safeguards, and their rights, including the option to withdraw at any time without consequences. All personal identifiers were removed, and data were pseudonymized during transcription to ensure confidentiality and privacy. Audio recordings were permanently deleted after verified transcription. These procedures ensured that the study was conducted in an ethical, transparent, and legally compliant manner.

### 2.6 Data collection methods

Prior to the FGDs, participants completed a brief pre-focus group questionnaire to collect sociodemographic and background information, including age, region of origin in Ukraine, duration of residence in Norway, living arrangements, use of devices and digital health, frequency of digital health use, and criteria for assessing the trustworthiness of online health information. These data were used to characterize the study population and contextualize the FGDs findings. Following the pre-questionnaire, FGDs were conducted using a semi-structured topic guide addressing health-seeking behaviors, barriers to care, experiences with DHIs, and cultural perceptions of health and healthcare. The semi-structured discussion guide included study-specific questions developed as part of the research, which are provided in Supplementary Appendix 1. For this paper, data related to participants' prior experiences with digital health, preferences regarding content, format, and language, perceived usefulness of DHIs for navigating healthcare services, and factors influencing continued use or discontinuation, were included in the analysis. FGDs were conducted in Ukrainian by a Ukrainian moderator in a neutral comfortable setting. This flexibility allowed participants to introduce topics they considered important, enriching the data. Each FGD lasted approximately 150 min, involved six participants, and was audio-recorded with participants' consent to ensure accuracy in transcription and analysis. After each FGD, participants were debriefed, and their questions or concerns were addressed, reinforcing the collaborative and respectful nature of the process.

### 2.7 Data collection instrument and technologies

The semi-structured guide was developed in English, translated into Ukrainian and back translated to ensure accuracy. This guide was used in the five FGDs. FGDs were audio recorded, and data were transcribed using Autoteskt, an artificial intelligence (AI)-based transcription and translation tool developed by the University of Oslo (39). Instruments did not require modification during fieldwork. Participant quotations were translated and lightly edited for clarity and readability (e.g., grammar and phrasing), while preserving their original meaning and intent.

### 2.8 Units of study

The study included thirty URW aged 18–55+. Five FGDs were conducted with six participants each, grouped by similar age: one group aged 18–24, two groups aged 25–54, and two groups aged 55+. This distribution enabled examination of generational differences relevant to ensuring cultural and age-appropriate insights for the study.

### 2.9 Data processing

Transcriptions produced by Autoteskt were cleaned by the research team and verified by Ukrainian interpreters through bilingual spot checks. Ambiguous terms were reviewed collaboratively to ensure consistency of meaning. Translation into English was conducted using Microsoft Word's translation tool following Ukrainian verification.

### 2.10 Data analysis

Data were analyzed using the reflexive thematic analysis (RTA) following Braun and Clarke's six-phase approach (40, 41). RTA was chosen as it supports the generation of interpretative themes as analytic constructions rather than categories emerging directly from the data. Inductive coding was conducted independently by two researchers with design research backgrounds. Codes and interpretations were subsequently compared and discussed to support reflexive engagement with the data. The data were then organized using Jesse James Garrett's Elements of User Experience framework (42). This framework was used to examine how healthcare experiences, system differences, and age-related patterns of digital engagement shape interactions with digital health. It also supported the translation of findings into user-centered design requirements. The framework offers a layered and systematic approach to understanding user experience, moving from abstract concepts to concrete interface elements, and enables a coherent structuring of the analysis. While widely applied in digital and design research, its use in healthcare remains limited; however, studies in related fields have used it alongside user-centered design and design thinking approaches to structure user needs and translate them into actionable design elements (43, 44). The analysis focused on identifying user-centered requirements to inform the design of accessible and context-responsive DHIs for URW in Norway, while also generating insights applicable to designing digital health in comparable settings.

### 2.11 Techniques to enhance trustworthiness

Trustworthiness was achieved through researcher triangulation, involving two analysts, reflexive memo writing, analytic documentation and bilingual spot checks by a Ukrainian native speaker to ensure translation integrity and coherent interpretation of the data.

## 3 Results

This section presents the results from the pre-focus group questionnaire and the FGDs. Questionnaire results focus on participants' characteristics, while FGDs findings are presented across nine themes derived from the data analysis.

### 3.1 Participants characteristics

**Figure 1:**
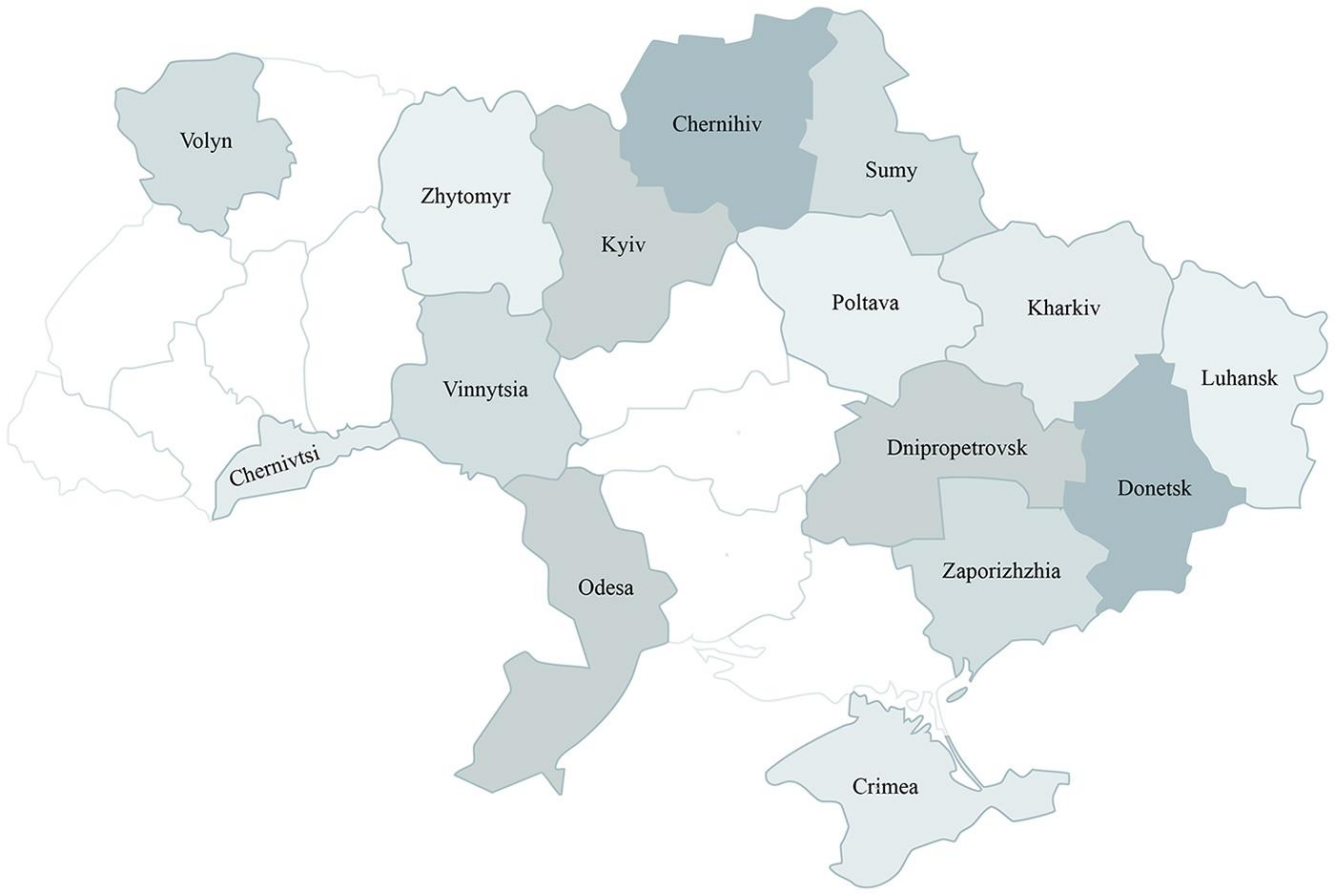
Map showing the Ukrainian oblasts represented in the study.

In total, thirty URW aged 18–72 years participated in the study, distributed across five FGDs: FG1 (18–24 years), FG2 and FG3 (25–54 years), and FG4 and FG5 (55 years and older). Findings from the pre-focus group questionnaire indicated varied lengths of residence in Norway, reliance on mobile devices for health information, and heterogeneous exposure to digital health, providing context for participants' discussions. Participants originated from Crimea and 14 oblasts of Ukraine and represented diverse professional backgrounds, reflecting geographical, cultural, and educational diversity (Figure 1). Length of residence in Norway ranged from 3 months to the onset of the conflict, with an average stay of 2.3 years. Sociodemographic characteristics of the participants are presented in Table 1, while patterns of digital health tool use and sources of health information by age group are summarized in Table 2.

**Table 1:** Sociodemographic characteristics.

Age group (years)	Focus group	No. of participants	Mean age (years)	Mean length of stay in Norway (years)	Living arrangement
18–24	1	6	20.67	2.74	(3) 50% Living alone (1) 16.7% Living with other family/relatives (2) 33.3% Renting a room in co-living
25–54	2	12	40.17	2.35	(6) 50% Living with partner and children (2) 16.7% Living with partner (3) 25% Living with children (1) 8.3% Living alone
55 years and older	2	12	64.17	2.10	(3) 25% Living with partner (2) 16.7% Living with children (4) 33.3% Living alone (3) 25% Living with other family/relatives

**Table 2:** Digital health tool use and sources of health information by age group.

Age group (years)	Devices used to look for health information	Frequency of digital health tool use in Norway	Digital Health Used	Trustful digital sources of health information
18–24	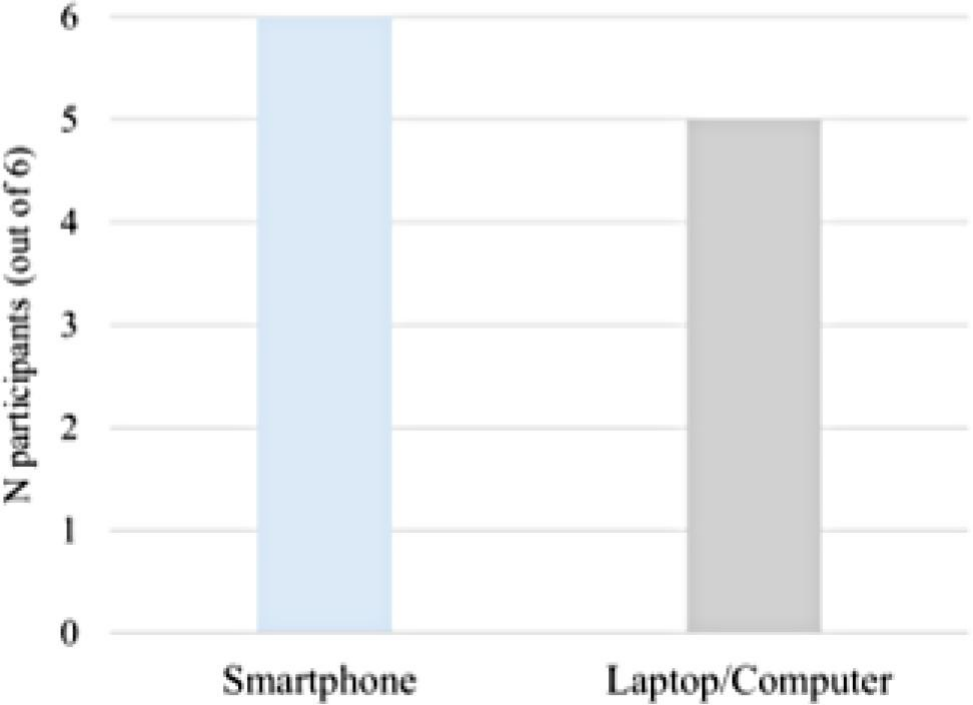	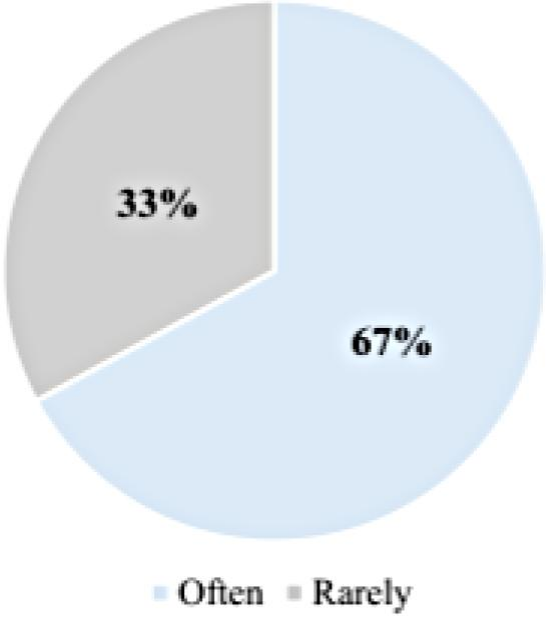	- Helsenorge - Helsami - Norwegian Directorate of Health - Google - Ung.no - ChatGPT	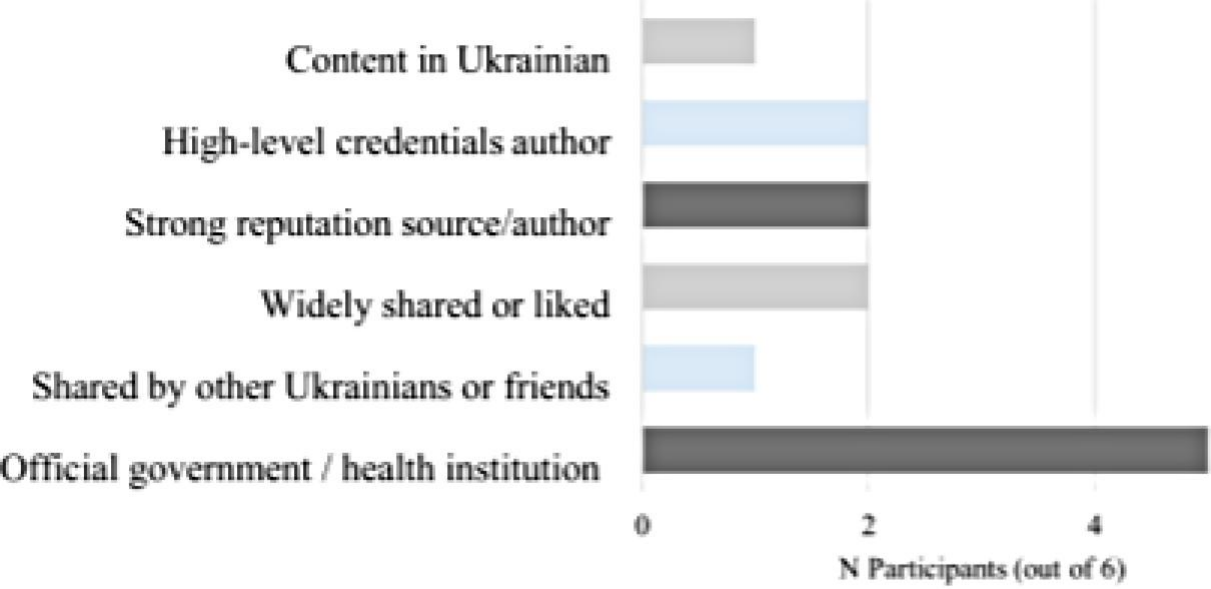
25–54	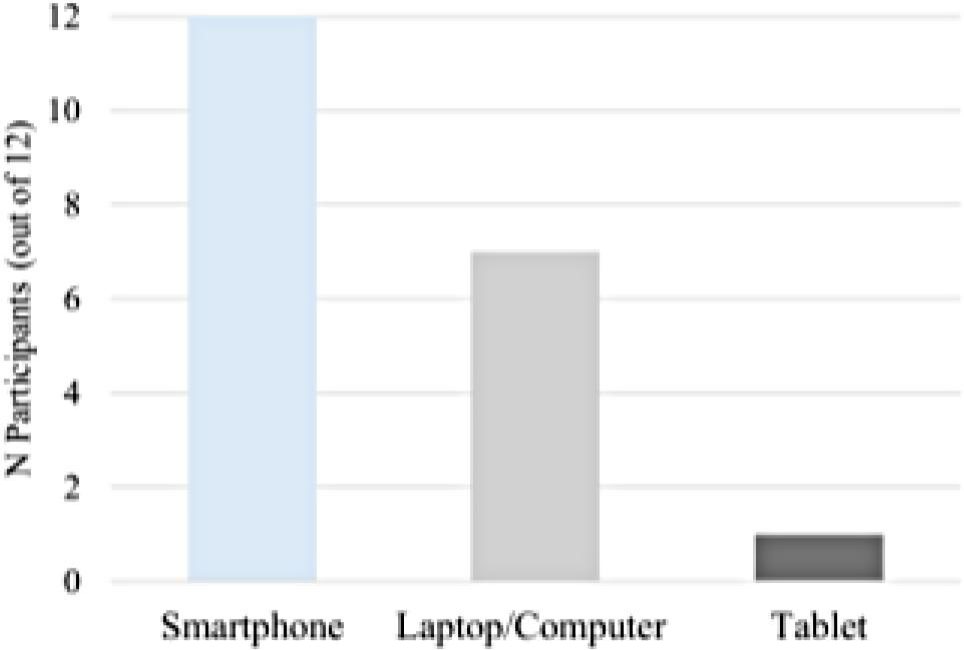	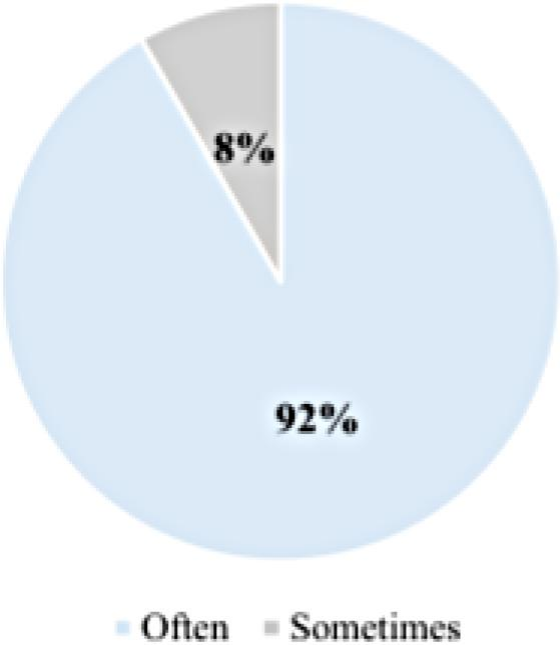	- Helsenorge - Helsami - Google - ChatGPT	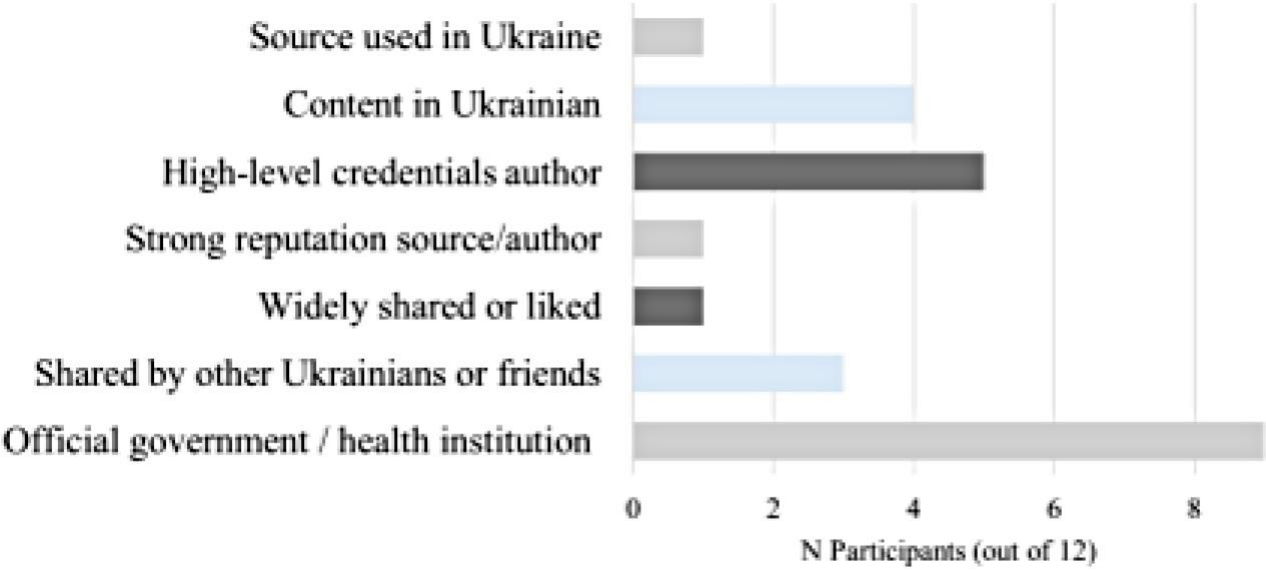
55+	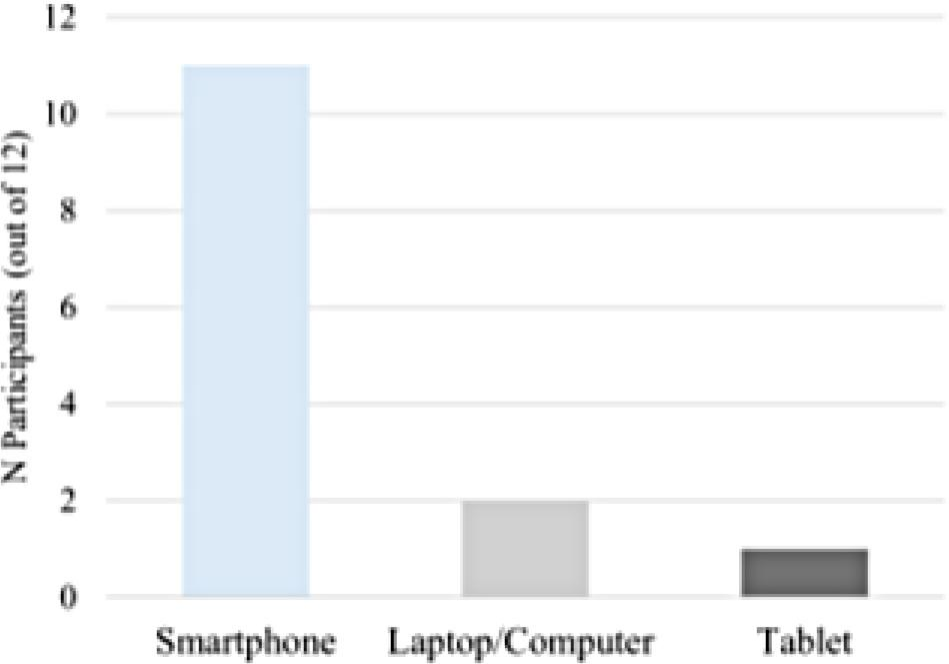	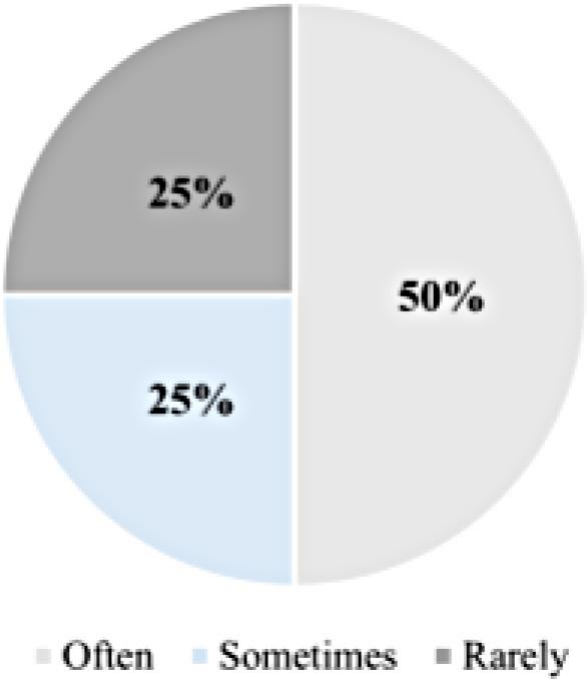	- Helsami - Google - Digipost - Helsenorge	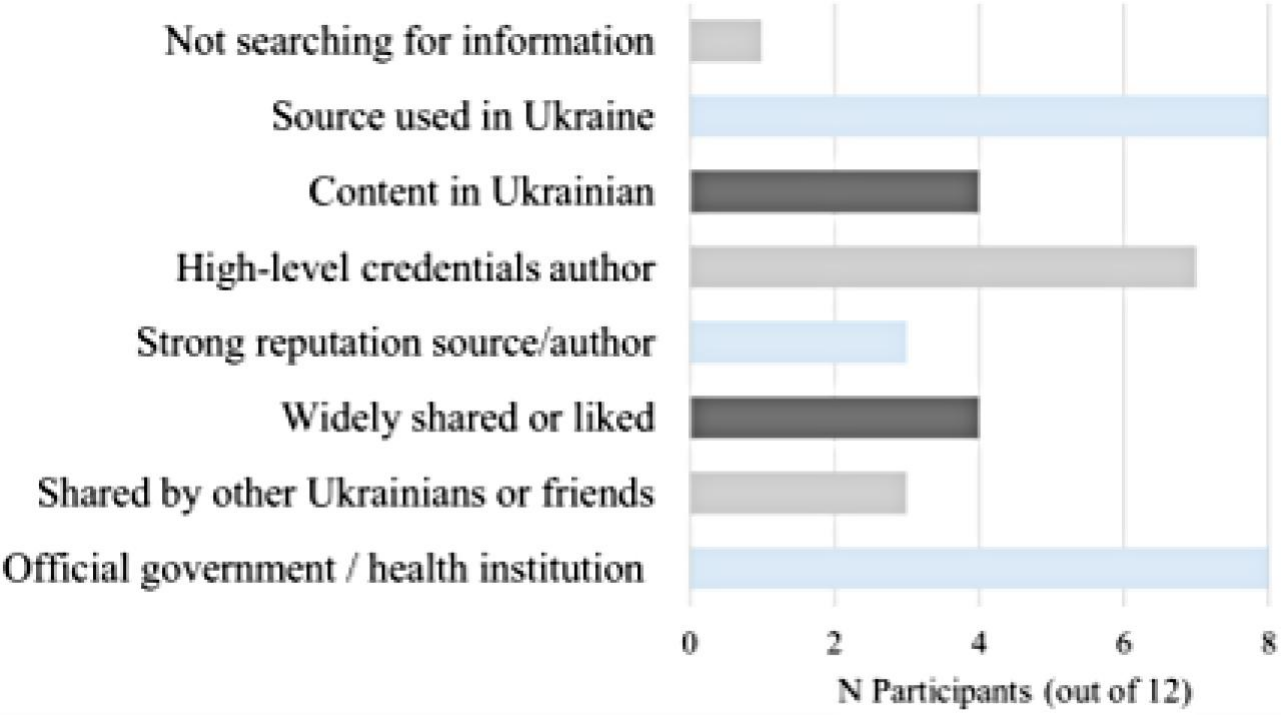

### 3.2 Focus group discussions thematic findings

**Figure 2:**
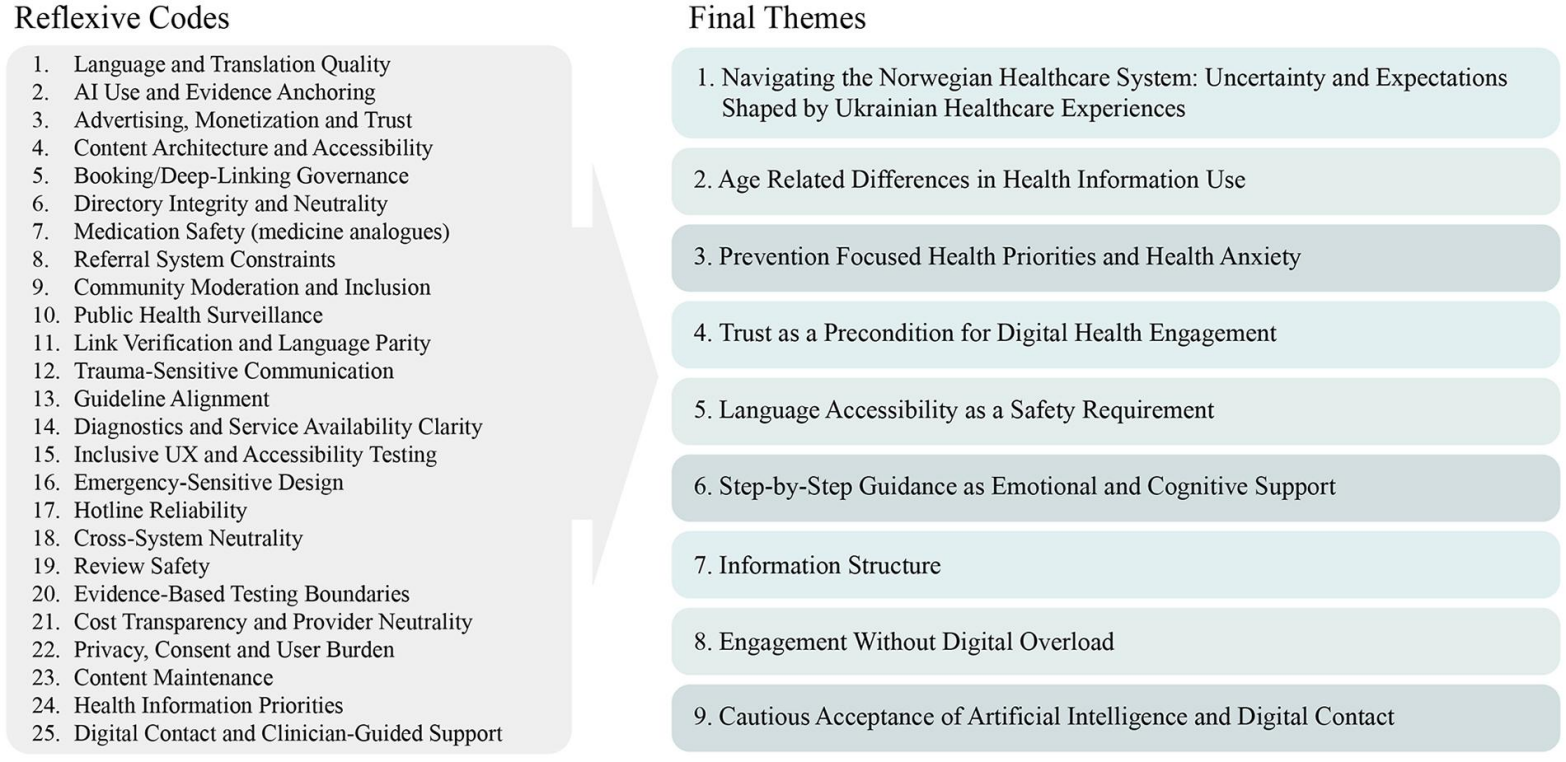
Consolidation of the final nine themes from the 25 reflexive codes.

The analysis generated 25 reflexive codes, which were consolidated into nine overarching themes. These themes describe participants' experiences of healthcare navigation formed by prior system experiences, age-related differences in information-seeking practices, prioritization of preventive health, the role of trust in engagement, language accessibility, the need for step-by-step guidance, the influence of information structure and format on usability, the effects of information overload on disengagement, and the use of artificial intelligence for health information. Figure 2 illustrates the relationship between the reflexive codes and the overarching themes.

#### 3.2.1 Theme 1: Navigating the Norwegian healthcare system: uncertainty and expectations shaped by Ukrainian healthcare experiences

Participants described navigating the Norwegian healthcare system through the lens of prior knowledge and experiences from Ukraine. Differences between the two systems contributed to uncertainty about how to access and use healthcare services. Participants reported difficulties understanding where to seek care, whom to contact, associated costs, and the steps required, particularly in relation to referrals, specialist services, examinations, and urgent situations. This uncertainty and mismatch in expectations were frequently associated with feelings of confusion, frustration, and a reduced sense of control.

Among participants aged 18–24, navigation challenges were primarily described as difficulties in orienting themselves within an unfamiliar system. The absence of informal family guidance typically relied upon in Ukraine, increased uncertainty in decision-making. One participant explained: “*The system is different from the Ukrainian one, and you cannot ask for help from your mother. I would like to have information about what services I have access to, or where I can get them*” (Ukrainian woman, 18–24 years).

Participants aged 25–54 expressed frustration with referral pathways that required repeated visits to a general practitioner (GP) before accessing specialist care, whereas in Ukraine direct access to specialists was possible. One participant noted, “*In Ukraine we can just sign up directly… here everything is slow*” (Ukrainian woman, 25–54 years FG-1). The need for information about Ukrainian or Russian-speaking healthcare professionals was also emphasized: “*There is a very large demand in the group for Russian and Ukrainian-speaking doctors, and medical workers in general*” (Ukrainian woman, 25–54 years FG1), and “*I would like online consultations with Ukrainian doctors*” (Ukrainian women, 25–54 years FG2).

Participants aged 55-years-and-older described these contrasts more critically, particularly in relation to accessing service listings, booking appointments, and the lack of online consultations, reviews and ratings. Such services were perceived as absent in Norway, contributing to frustration among URW. Two participants stated: “*I would like, just as in Ukraine, to have information of the clinics, the doctors, their bosses, addresses, prices*” and “*In Ukraine, it is not a problem to make an appointment*” (Ukrainian women, 55 + years FG1).

#### 3.2.2 Theme 2: Age-related differences in health information use

Age-related differences were observed in participants' use of and preferences for health information. These differences shaped information needs related to the Norwegian healthcare system, emergency care, medications (over-the-counter and prescription), specialist services, and preventive health.

Participants aged 18–24 stressed the need for clear explanations of how the Norwegian healthcare system functions, including how to access services and specialists. They highlighted the importance of easily accessible contact information and guidance on when and where to seek care. Two participants stated: “*Phone numbers of different hospitals with the services and expenses are needed, and perhaps some doctors that you can contact in certain situations*” and “*We also need direct information about how the Norwegian system works: how to reach specialists and what opportunities exist*” (Ukrainian women, 18–24 year).

Participants aged 25–54 expressed similar needs but placed additional emphasis on information about Ukrainian- and Russian-speaking healthcare professionals, particularly gynecologists. They also underscored the need for information on medications, including equivalents between Ukrainian and Norwegian drugs and whether prescriptions are required. One participant explained: “*Make a table of equivalent medicines, indicating which require a prescription and which are over-the-counter. I currently find this information on social networks from people who have lived here for a long time, but it is not practical to keep taking screen*” (Ukrainian woman, 25–54 years FG2). Participants also underscored the need for accessible information in urgent or stressful situations: “*You need a source that when you are in a state of panic attack, or you are nervous, you can go in and quickly find this information*” (Ukrainian woman, 25–54 years FG2).

Participants aged 55-years-and-older underlined the need for information on preventive care, types of medication and their analogs, costs associated with doctors and clinics, and updates on medical services and practices. One participant said, “*There should be a general site where you should be able to find a price list, the description of doctors with their titles and so on, so we can navigate and choose a clinic and a doctor*” (Ukrainian woman, 55+ years FG1). This underscores the importance of accessible specialist information and transparent pricing to support informed healthcare decision-making.

#### 3.2.3 Theme 3: Prevention-focused health priorities and health anxiety

Participants consistently underscored prevention as a central health concern, particularly in relation to cancer screening, women's health, vaccination, chronic illness, and mental health.

Participants aged 18–24 focused primarily on information related to oncology, gynecology and psychological support. One participant explained: “*Information about cancer is very important for Ukrainians, because we have a lot of cases. We have Chernobyl. We are very prone to oncology. I read that cervical cancer is very common in Ukraine*” (Ukrainian woman, 18–24 years). Awareness of early warning signs was also accentuated, particularly the need to distinguish between serious and non-serious symptoms: “*I want to understand what is dangerous and what is not*” (Ukrainian woman, 18–24 age group).

Participants aged 25–54 highlighted the need for information on age-related physiological and hormonal changes. One participant stated: “*I want from a medical point of view an explanation of how a woman's body changes with age. How the hormonal background and physical condition changes. Because I don't know how my hormones change*” (Ukrainian woman, 25–54 years FG2). Concerns related to caregiving responsibilities were also prominent: “*We are all basically mothers, we think about our children… And yes, I don't want to get sick*” (Ukrainian woman, 25–54 years FG2). Participants in this group also referred to missed screenings and difficulties accessing preventive services.

Participants aged 55-years-and-older underlined preventive health information related to chronic diseases, infectious diseases, vaccination, and screening programs. Emerging and seasonal illnesses were described as important concerns. One participant explained: “*We need to know which diseases are in the city now, the flu or some COVID that is already new… This information is also needed. It may be necessary to get vaccinated*” (Ukrainian woman, 55+ years FG 2).

#### 3.2.4 Theme 4: Trust as a precondition for digital health engagement

Trust was consistently described as an important factor influencing participants' engagement with digital health resources. Participants identified several features that affected their level of trust, including advertising and spam communication, unverified sources, lack of moderation, misleading information, and outdated content.

Participants aged 18–24 expressed strong negative reactions to advertising-driven platforms and intrusive formats. One participant stated: “*I directly delete apps that have full screen advertisement where you have to wait before closing them*” (Ukrainian woman, 18–24 years). Distrust was also associated with pop-ups or sponsored content. However, some participants noted that informational messages could be acceptable if perceived as relevant: *“If the message contains useful health information, it can be interesting*” (Ukrainian woman, 18–24 years).

Participants aged 24–54 emphasized the importance of moderation, verified information and safety, particularly within digital community spaces. One participant explained, “*What would completely discourage me from using it is if people share false information in the chat*” (Ukrainian woman, 25–54 years FG2). Although reviews, ratings and comments were frequently mentioned as preferred formats, some participants expressed reservations, perceiving them as subjective and not always reliable: “*I do not think they are entirely objective… People are different, and the approach is different. Therefore, I cannot say unequivocally about the comments that I believe one hundred percent*” (Ukrainian woman, 25–54 years FG2).

Participants aged 55-years-and-older placed greater trust in recognized authorities and healthcare professionals. They also described perceived pressure or imposition of information as a source of distrust. One participant noted: “*Imposition will make me stop using a DHI*” (Ukrainian woman, 55+ years FG1), and “*I would prefer to hear from specialists… Not instructions, but recommendations on what to do in specific conditions*” (Ukrainian woman, 55+ years FG2).

#### 3.2.5 Theme 5: Language accessibility as a safety requirement

Participants described the use of the Ukrainian language as an important requirement for engaging with health information, particularly for understanding medical terminology and during consultations.

Participants aged 18–24 who were proficient in English or Norwegian generally reported less need for Ukrainian translation. However, one participant highlighted its importance: “*It is very important that information is available in Ukrainian*” (Ukrainian woman, 18–24 years).

Participants aged 25–54 stressed a stronger need for health information in Ukrainian. Machine-only translations were described as difficult to understand, particularly in relation to medical terminology. One participant explained: “*It would be better if this website was available in Ukrainian, because translations often make things much more complicated, especially with medical terminology*” (Ukrainian woman, 25–54 years FG1).

Participants aged 55-years-and-older described the acceptability having both Ukrainian and Norwegian language options: “*Information can be Norwegian, but there should be a translation*” (Ukrainian woman, 55+ years FG2).

#### 3.2.6 Theme 6: Step-by-step guidance as emotional and cognitive support

Participants expressed a need for clear, step-by-step guidance outlining what actions to take in specific health situations.

Participants aged 18–24 described the need for guidance on how to navigate the Norwegian healthcare system and how to respond to different symptoms. Two participants stated: “*We should know which symptoms require attention and when to contact a doctor*”, and “*We need information about how the system works, including how to access specialists, and what options are available*” (Ukrainian women, 18–24 years).

Participants aged 25–54 pointed out practical guidance for decision-making, particularly in urgent or out-of-hours situations. They described a need for clear instructions on how to respond to specific health conditions. Participants noted: “*We need clear instructions on what to do in specific situations—for example, if blood pressure is high, where to go, who to call, and what steps to follow*”, and “*It is especially important to know what to do in urgent situations, for example at night or early in the morning*” (Ukrainian women, 25–54 years FG1).

Participants aged 55-years-and-older described similar needs for practical, action-oriented information, particularly in urgent situations: “*We need useful information on what to do in different conditions. If something happens, where should we go? The most important thing is where to go, how quickly, and how they will react to it*”, and “*People need to put it into practice… information should be practical and applicable*” (Ukrainian women, 55+ years FG2).

#### 3.2.7 Theme 7: Information structure

Participants described the structure and presentation of information as important factors influencing usability. Preferences included clear organization, layered content, and easy navigation, while dense text and cluttered layouts were described as discouraging use.

Participants emphasized the value of combining multiple formats to support different preferences and needs. Two participants explained: “*For an application or website, it is better to combine podcasts, videos, and text, because people are different*”, and “*These formats should complement each other—if a video is not clear, I can read the text, and if I do not understand the text, I can look at visuals*” (Ukrainian women, 18–24 years).

Participants aged 18–24 described a preference for text-based content supported by audio and visual elements, such as podcasts or videos. One participant stated: “*I prefer text-based articles first. Podcasts or other formats can be useful as additional support*” (Ukrainian woman, 18–24 years). Participants in this group also referred to interactive tools, such as health tracking features and communication tools, including chat or chatbot functions: “*I imagine an application that tracks your health and, if there are concerns, provides a support contact*”, and “*It would be helpful if the information entered could be shared with doctors when needed*” (Ukrainian women, 18–24 years).

Participants aged 25–54 preferred concise text supported by visual elements and links to official or trusted sources: “*I also think that short texts with links to official sources would be helpful*” (Ukrainian woman, 25–54 years FG2), “*I think that text with visual support is important*” (Ukrainian woman, 25–54 years FG1) and “*Texts with images and links to reliable sources, where there are explanations or videos, are useful*” (Ukrainian woman, 25–54 years FG2). Participants also mentioned reminders, such as notifications about vaccinations or appointments, as useful formats: “*Reminders about vaccinations or upcoming appointments would be helpful*” (Ukrainian woman, 25–54 years FG1).

Participants aged 55-years-and-older described varied preferences depending on individual needs. Some preferred visual or audio formats due to difficulties with reading. Participants stated: “*I prefer videos because I can't see well and reading is very difficult for me*” (Ukrainian woman, 55+ FG1) and “*I am comfortable with content that can be listened to*” (Ukrainian woman, 55+ years FG2).

Participants also referred to individual differences in information processing: “*I have ADHD, and… it is difficult for me to read long texts*” (Ukrainian woman, 18–24 years).

#### 3.2.8 Theme 8: Engagement without digital overload

Participants described several features that discouraged continued use of digital health resources. These included excessive amounts of information, disorganized content, and intrusive interface elements.

Participants aged 18–24 described negative reactions to overloaded interfaces, pop-ups, and disruptive elements. “*When everything is overloaded, it's very difficult to focus*”, *and* “*If there are pop-up advertisements with sound, loud music… this application goes straight to the trash forever. The same if there is a lot of noise on the page, it distracts me a lot*” (Ukrainian women, 18–24 years).

Participants aged 25–54 identified additional usability issues, including small font sizes, excessive use of colors, poor spacing, and unclear navigation structures. One participant stated: “*If the website is too complex, it is difficult to find information in it, then I would not use it. It will be easier to find information through Google*” (Ukrainian woman, 25–54 years FG2).

Participants aged 55-years-and-older described the importance of organizing content into clear categories to avoid overload, “*It should be structured in sections, for example, one for doctors, another for diseases, etc*” (Ukrainian woman, 55+ years FG1).

#### 3.2.9 Theme 9: Cautious acceptance of artificial intelligence and digital contact

Participants expressed cautious openness and clear boundaries toward AI-supported tools and digital contact with clinicians. AI was generally considered acceptable for information seeking and initial orientation, but not for treatment or clinical decision-making.

Participants aged 18–24 described AI tools as convenient but not fully reliable. One participant stated: “*ChatGPT is useful, but it is not something I fully trust. I use it to ask questions, but I would not rely on it for treatment*” (Ukrainian woman, 18–24 years). Participants in this group reported using AI tools when information could not be easily found elsewhere. They also referred to the use of chatbots and symptom-tracking tools that provide automated suggestions and guidance: “*Imagine a tool that tracks symptoms, then provided you with warnings, and transferred to some links, articles, what can be done in such a situation, who to contact…*” (Ukrainian woman, 18–24 years).

Participants aged 25–54 expressed ambivalence toward AI, particularly in contexts where access to healthcare services was limited. For example, some participants described using AI tools as a substitute for unavailable services, such as psychological support in Ukrainian. One participant explained: “*Many of us need psychological help that we cannot get as there are no certified psychologists who could communicate in Ukrainian. I use ChatGPT as my personal psychologist. And this is also a controversial decision, because ChatGPT always agrees with you*” (Ukrainian woman, 25–54 age years FG 2).

Participants aged 55-years-and-older emphasized the importance of human interaction and professional guidance. Digital contact was primarily valued for communication with healthcare professionals, particularly through online consultations, “*I would like the option of online consultations with doctors, where I can ask questions and receive recommendations*” (Ukrainian woman, 55+ years FG2). Table 3 shows a summary from some of the themes about the digital health information needs across the different age groups.

**Table 3:** Digital health needs across age groups.

Age group	Health prevention content priorities	Navigation needs	UX/accessibility needs	Interaction/feature needs	Safety and trust needs
18–24	Gynecology, oncology, HPV/cervical screening, mental health	Visual maps, urgent vs. non-urgent symptoms, “Where to go quickly”, referral rules	Logic and structured interface, short clips, mobile-first	Trackers, chat guidance, symptom prompts, data-sharing with clinicians, updates	Source badges, verified comments, last-update stamps, no ads
25–54	Women's health, hormones, cancer screening, child vaccines	Detailed triage, referral rules, urgencies step-by-step guidance, public/private breakdown, medication equivalence	Expanders, hybrid text and video, checklists, clean interface, mobile-first	Reminders, quick booking links, online consultations	BankID-verified reviews, Ukrainian language, verified sources, cost transparency
55+	Chronic diseases, contagious diseases, influenza/pneumococcal vaccination, cancer screening	Urgencies step-by-step guidance, audio instructions, simple navigation flows, medication equivalence, referral rules	Large fonts, high contrast, captions, audio, mobile-first	Voice/video orientation, one-tap actions, online consultations	Official doctor content, cost transparency, clear red-flag instructions, Ukrainian language, no imposition

## 4 Discussion

This study explored how URW's prior healthcare experiences shape their navigation of the Norwegian healthcare system, as well as their preferences and challenges in accessing health information and engaging with DHIs. The nine consolidated themes provide insight into the user-centered requirements for a supportive DHI and form the basis of a refugee-centered, design-informed framework. The findings indicate that challenges in digital health navigation are not primarily driven by a lack of health information, but by unmet expectations, age-specific differences in information use, limited trust in information sources, language inaccessibility, the absence of clear step-by-step guidance, and unstructured or cognitively overwhelming complex interfaces.

Insights from the FGDs highlight the importance of including diverse age groups namely young, middle-aged, and older women. This generational diversity revealed differentiated patterns in how digital health information is accessed, interpreted, and acted upon, as well as age-specific preferences in content and format. Rather than representing distinct user categories, these findings suggest overlapping and coexisting practices that should be addressed through inclusive and accessibility-oriented design approaches.

### 4.1 Population-level patterns of digital health intervention use and information sources

Findings from the pre–focus group questionnaire further contextualize these results, demonstrating widespread and frequent use of digital health information across all age groups. Unlike many other refugee populations, Ukrainians can be considered digitally experienced, given their familiarity with technology and the highly digitalized healthcare system in Ukraine. A study in Hungary similarly reports high levels of digital literacy and active engagement with digital tools among URW (44).

Smartphones emerged as primary access device, and national platforms such as Helsenorge were consistently used, often alongside general search engines such as Google. Despite this high level of engagement, participants reported difficulty translating digital information into concrete healthcare actions within the Norwegian system. This suggests that while digital engagement predated the FGDs, it did not necessarily result in effective healthcare navigation.

**Figure 3:**
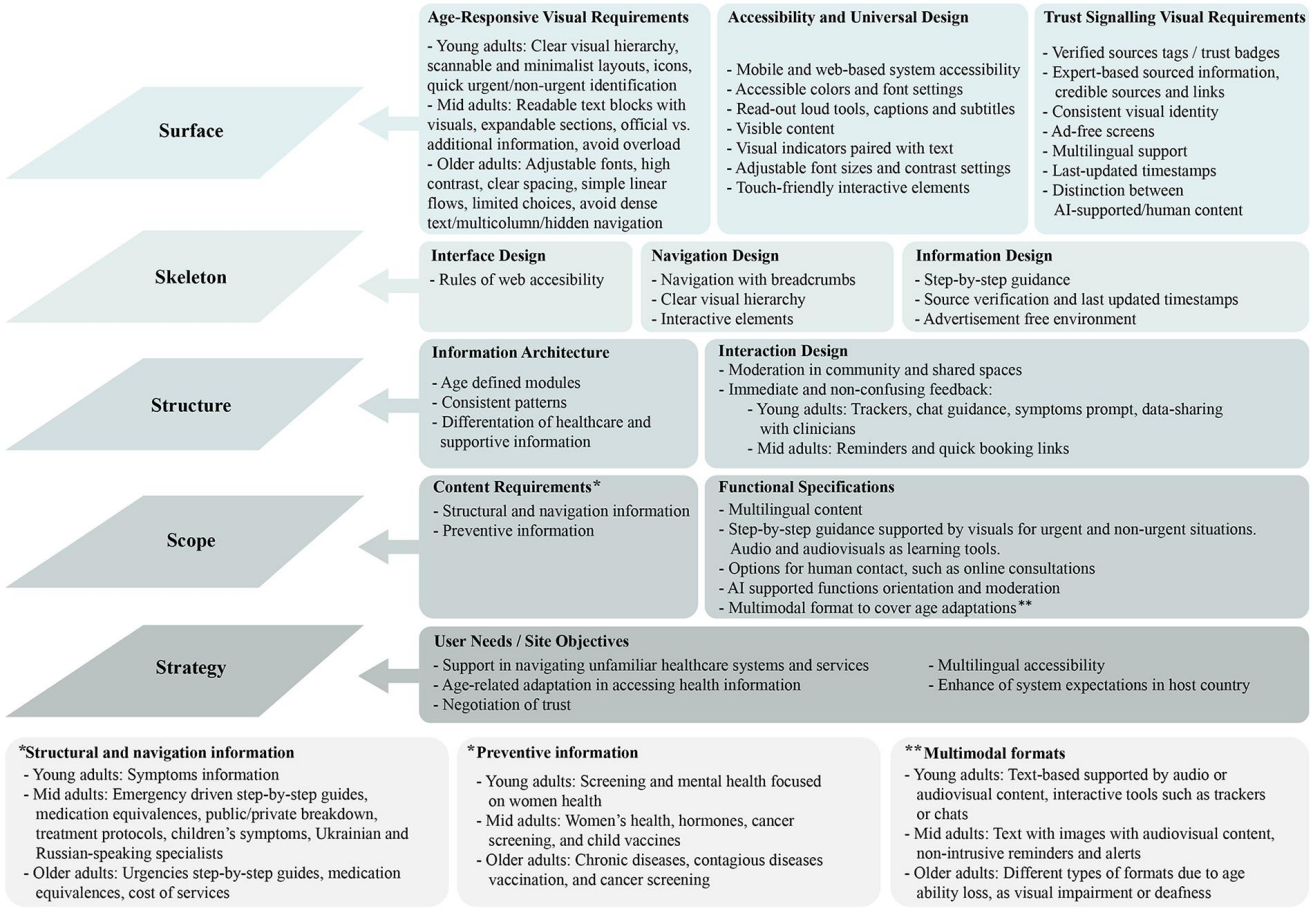
User-centered requirements for digital health interventions for Ukrainian refugee women.

Differences in usage patterns were also evident across age groups. Younger and middle-aged participants reported broader engagement with diverse digital sources, including AI-based tools and informal platforms, whereas older participants relied more heavily on official services such as Helsenorge and Digipost. These patterns indicate that digital health already functions as an entry point to healthcare information but does not adequately support users in interpreting pathways, responsibilities, or next steps. Age-related differences identified in this study have direct implications for the design of digital health interventions. Younger users demonstrated preferences for interactive and dynamic features, while middle-aged users emphasized structured and actionable information, and older users required simplified interfaces and accessibility-oriented features. Importantly, these differences do not necessitate separate systems, but rather adaptable design solutions within a unified platform. This includes multimodal content, flexible navigation structures, and customizable interface elements that support multiple user pathways. Additionally, these age-specific requirements are explicitly operationalized within the proposed framework and detailed in Figure 3. This demonstrates how age-related differences can be translated into implementable design components within the information architecture.

Taken together, these findings highlight a gap between digital access and digital support. Existing systems provide information but do not function effectively as navigational or decision-support infrastructures. Addressing this gap requires a shift from information provision toward structured, trustworthy, and action-oriented digital health design.

### 4.2 Emerging patterns in current digital health intervention access and navigation among Ukrainian refugee women

The findings position DHIs as supportive and mediating tools rather than purely informational resources. Participants' concerns about where to go, whom to contact, and what actions to take were frequently described as situational, emotionally charged, and context-dependent. This uncertainty was particularly pronounced in time-sensitive or preventive contexts. Differences between the Ukrainian and Norwegian healthcare systems emerged as a central factor influencing these experiences, creating systemic barriers to navigation and understanding.

In Ukraine, the historically specialist-oriented system allowed relatively direct access to healthcare providers and medications (45). In contrast, the Norwegian system follows a GP-centered model (46), where general practitioners act as gatekeepers to specialist services. These structural differences contributed to confusion and frustration among URW when attempting to access care. Additionally, ongoing digital health reforms in Ukraine, including the prioritization of a unified e-health system since 2017 (47), have shaped expectations that do not align with the Norwegian digital health infrastructure. These challenges may not be interpreted solely as difficulties in adapting to a new system. Rather, they also reflect expectations shaped by prior experiences with digitally integrated healthcare services, including transparency, accessibility, and responsiveness. In this sense, participants' concerns may indicate opportunities for improvement in host-country systems.

Trust emerged as a key factor influencing engagement with both DHIs and healthcare systems. Evidence from previous research indicates that trust is a significant barrier for Ukrainian refugees accessing healthcare services (48), and broader studies show that migrants often experience mistrust due to unmet expectations or prior negative experiences (49). Within DHIs, sources of distrust include advertising, outdated or unverified information, machine-only translations, lack of moderation, and concerns about data privacy (50). Trust in this study operates across multiple, interconnected dimensions. First, participants expressed concerns regarding the credibility of digital information sources, including issues such as misinformation, lack of verification, and advertising. Second, trust was closely linked to perceptions of healthcare institutions, particularly in relation to system transparency, accessibility, and responsiveness. Third, prior experiences with the Ukrainian healthcare system shaped expectations, influencing how participants evaluated both digital tools and healthcare pathways in Norway. These overlapping dimensions indicate that trust is not solely a feature of digital design but is embedded within broader socio-technical and migration-related contexts.

These dimensions are translated within the proposed framework into specific design considerations, including source verification, moderated interaction spaces, professional language translation, transparent system alignment, and the minimization of advertising, thereby operationalizing trust as a designable component rather than an abstract concept, that can be seen in Figure 3.

These findings suggest that trust must be embedded at the level of system design and governance. Advertisement-free environments, professional translation, transparent sourcing, moderated interaction spaces, and visible alignment with public healthcare institutions should be considered foundational requirements rather than optional features. Prior studies similarly emphasize the importance of professional translation and verified information in designing digital health resources for migrant populations (51).

Although participants broadly agreed on key information needs such as system navigation, preventive care, and service access they differed substantially in how this information should be delivered. Concise text was preferred in urgent situations, whereas multimedia formats were considered more suitable for learning and ongoing engagement. This variation highlights the importance of multimodality in ensuring accessibility across age groups and abilities, consistent with United Nations guidelines on disability-inclusive communication (52).

Participants' responses to dense text, cluttered layouts, broken links, and intrusive notifications further demonstrate that information structure has both practical and emotional consequences. Poorly organized content increased frustration and disengagement, whereas structured and predictable layouts supported confidence and continued use. Step-by-step guidance was particularly valued, not only for its clarity but also for its calming effect during stressful situations. These findings align with prior research accenting the importance of concise and carefully structured information (26). Together, they position information architecture as central to both cognitive processing and emotional regulation, reinforcing usability and accessibility as safety-critical design considerations.

Participants also expressed cautious openness toward AI-supported features and digital communication with healthcare professionals. AI tools were often used as secondary or fallback sources when official systems failed to provide timely or clear information but were not trusted for diagnostic or decision-making purposes. Younger and middle-aged participants reported more frequent use of AI for information seeking, whereas older participants prioritized human reassurance and professional contact.

These findings suggest that AI should function as a supportive and assistive component within DHIs, combined with clear disclaimers, transparent functioning, and visible pathways to professional care. Evidence indicates that AI-based tools currently have limitations in meeting complex health needs among refugee populations (53), reinforcing the importance of human involvement. AI integration should therefore be approached cautiously, particularly given concerns related to reliability, validation, and cultural sensitivity (54). Human-guided support remains essential, especially in situations involving uncertainty or perceived health risks.

### 4.3 User-centered requirements for digital health interventions for Ukrainian refugee women

To translate the findings into actionable design guidance for DHIs, this study draws on Jesse James Garrett's Elements of User Experience framework (42) as an interpretive lens. The framework provides a hierarchical structure that connects abstract user needs to concrete system design and interface elements determining URW experiences. The identified themes were mapped across the five information architecture layers (strategy, scope, structure, skeleton, and surface) to systematically link user needs with design considerations.

The resulting requirements offer a coherent, design-informed foundation for developing DHIs, conceptualized here as digital health platforms through which health information is accessed, interpreted, and acted upon within healthcare systems. Figure 3 summarizes these user-centered requirements and illustrates how qualitative insights are operationalized within the information architecture framework adapted from Jesse James Garrett (42).

#### 4.3.1 Strategy

The strategy layer defines the overall purpose of the system, including user goals and system objectives. At this level, the findings highlight healthcare navigation and access to reliable information as the primary user goals. Further, across all ages, preventive information constituted a significant barrier to accessing the services demanded by these women. These findings informed the definition of the overall objectives of DHIs to support navigation within unfamiliar healthcare systems while addressing age-related access preferences, trust considerations, multilingual accessibility, and alignment with system expectations in the host country. Additionally, the DHI should include preventive health practices and efforts to manage health related anxiety, particularly through early awareness, screening, and vaccination information. These findings are consistent structural differences between the Ukrainian and Norwegian healthcare systems, combined with participants' prior experience in a highly digitalized environment, contributed to unmet expectations (55). In the Norwegian context, gaps were identified in digital service provision, including limited access to online consultations, difficulties booking appointments when interpreters are required, and the absence of integrated information about healthcare providers and services (55).

#### 4.3.2 Scope

The scope layer defines the content and functionality of the system, including what information is provided and how it is delivered (42). Across all age groups, participants emphasized the need for both structural healthcare information and preventive health content. Structural information needs were largely consistent, including system navigation, GP referrals, access to specialists, and transparency regarding available services. In contrast, preventive health priorities varied by age. Younger participants focused on symptoms, mental health, and oncology awareness. Middle-aged participants underlined emergency guidance, medication (including equivalents), public–private system differences, treatment protocols, children's health, and access to Ukrainian- or Russian-speaking providers. Older participants underlined cost transparency, medication information, and emergency guidance.

These findings are consistent with previous European research reporting widespread difficulties among Ukrainian refugees in accessing structural healthcare information (12). They also reinforce the need for integrated, multi-topic DHIs rather than single-issue interventions (18–20, 25). From a functional perspective, content delivery must be multimodal and responsive to age-related preferences. Younger users preferred text supported by audiovisual and interactive elements, while middle-aged users favored structured text with visual support. Older users required adaptable formats due to sensory and functional constraints. These patterns align with existing research on culturally adapted digital health tools for refugee populations (21–23, 56). Interactive features, including AI-supported tools, require careful moderation, verification, and clearly defined safety boundaries to ensure appropriate and trustworthy use.

#### 4.3.3 Structure

The structure layer addresses how the system is organized and how users interact and navigate with its logic and flows, encompassing both information architecture and interaction design. Participants consistently emphasized the importance of logical and modular structure. Such modularity supports learning, reduces cognitive load, and facilitates navigation, as demonstrated in prior DHI research (24, 25). Suggested organizational strategies included categorization by health topic, user group, or system function (e.g., navigation, prevention, emergencies). Consistency across these categories is essential to support usability and scalability. Interaction design features were also underscored, particularly the importance of feedback mechanisms. Immediate system feedback enhances navigation and reduces uncertainty (57). Age-related differences were evident: younger participants preferred interactive tools such as trackers and symptom prompts, middle-aged users valued reminders and appointment links, and older users required simplified interactions supported by voice or video guidance. While community and chat-based features were viewed positively, participants stressed the need for moderation to prevent misinformation and ensure safe engagement.

#### 4.3.4 Skeleton

The skeleton layer defines how information is presented, how users navigate the interface, and how elements are structured on screen. It focuses on interface design, navigation, and information layout. Participants emphasized the need for clear visual hierarchy and intuitive navigation, supported by structured elements such as breadcrumbs. Accessibility features including captions, translated audio, visuals, and readable text are essential to support different user needs. Interactive elements, such as avatars and guided navigation tools, can enhance usability by supporting user orientation within the system (25, 27). Importantly, step-by-step guidance remains a core requirement, providing both cognitive clarity and emotional reassurance during decision-making.

Trust-related visible features were also highlighted, including source verification, timestamps, and up-to-date information, which increase reliability. Conversely, advertisements, pop-ups, and excessive notifications were identified as disruptive and negatively impactful for trust and usability. Previous research confirms that such elements can contribute to information overload and reduced trust in health systems (58).

#### 4.3.5 Surface

The surface layer represents the visual design of the system, including layout, typography, color schemes, and overall visual identity. These elements directly influence usability, accessibility, and trust. Age-responsive and accessibility-driven design principles are particularly important at this level. Younger users preferred minimalist layouts with strong visual hierarchies and intuitive icons supporting rapid decision-making. Middle-aged users favored structured layouts with visual support and expandable content that allows control over information depth. Older users required larger fonts, high-contrast visuals, simplified navigation flows, and audio support due to sensory limitations. Universal design principles are essential, including adaptable interface settings, touch-friendly interactions, and clear visual indicators. Features such as read-aloud tools, captions, and adjustable color schemes further enhance accessibility (26, 27). Mobile-first and responsive design is also critical, given the widespread reliance on smartphones.

Finally, visual trust signals play a central role in engagement. These include consistent visual identity, verified content labels, credible sources, multilingual presentation, and visible update timestamps. Language and cultural adaptation remain central to effective DHI implementation for refugee populations (28). Advertising should be minimized, and AI-supported features must be transparent and supported by human oversight.

### 4.4 Proposed refugee-centered framework for designing digital health interventions for Ukrainian refugee women

**Figure 4:**
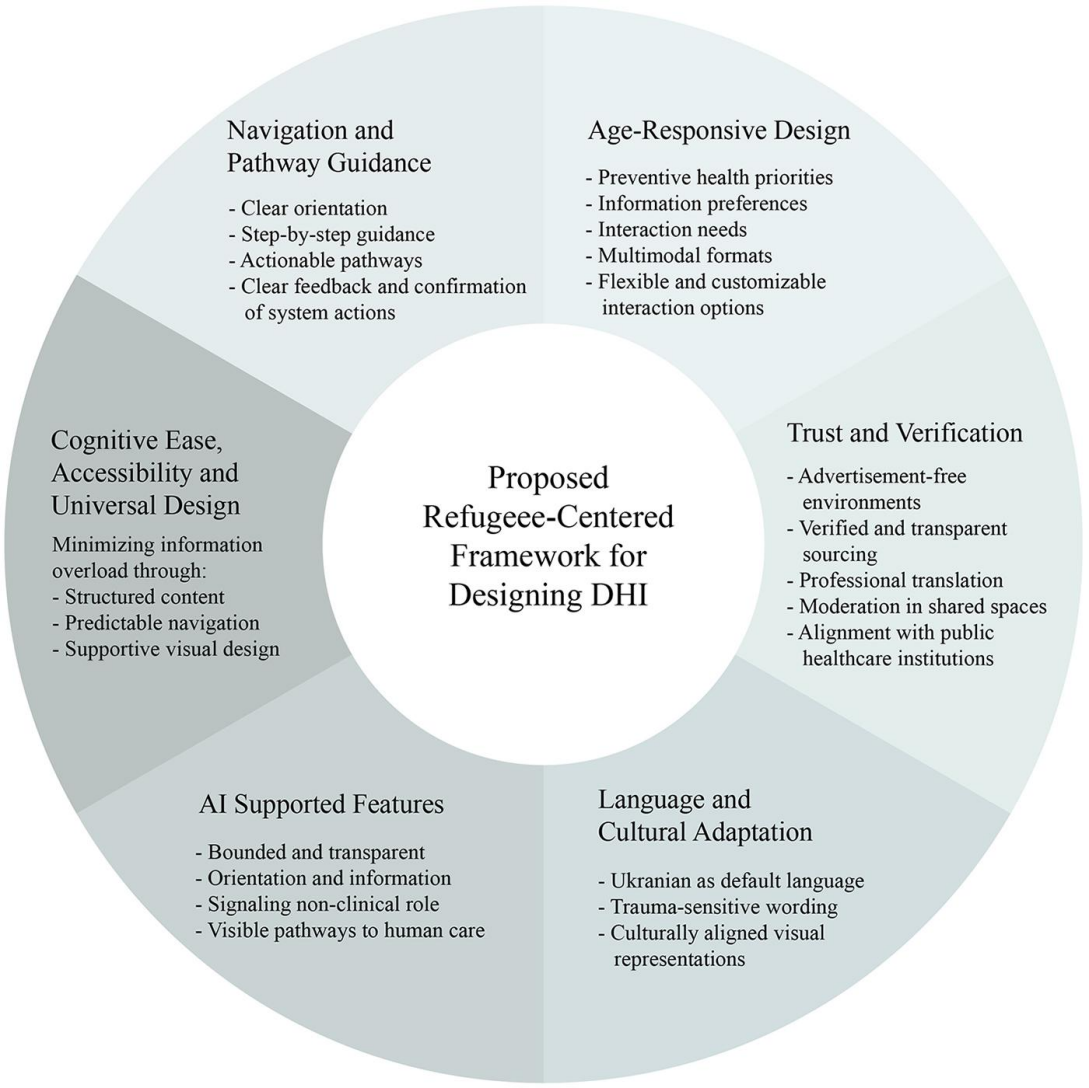
A proposed refugee-centered framework for design of digital health intervention (digital health platforms) for Ukrainian refugee women.

Building on the user-centered requirements for DHIs for URW (Figure 3), a domain-specific design framework (Figure 4) is proposed to guide the design of DHIs. The framework translates qualitative insights into structured, domain-specific design requirements that address key challenges of healthcare navigation in a migration context, including trust, language accessibility, system differences, and age-related patterns of digital engagement. Rather than introducing new design principles, the framework extends existing design approaches by operationalizing them within a specific socio-technical and healthcare context. In doing so, it provides actionable guidance for the development of digital health interventions tailored to refugee populations, positioning the framework not as a general methodology, but as a contextually grounded extension that specifies what matters for a particular user group and setting.

While the framework draws on established principles from user-centered and human-centered design, which primarily guide how to design with users, rather than what to design (59, 60), its contribution lies in defining context-sensitive design requirements derived directly from the lived experiences of URW. This aligns with experience-based design approaches in healthcare, which emphasize the importance of translating patients' lived experiences into actionable improvements in service design (57), as well as prior work adapting DHIs for refugee populations through human-centered design approaches (25).

The proposed framework operates in conjunction with the information architecture presented in Figure 3, where user requirements are systematically articulated across layers of design. While Figure 4 provides a conceptual synthesis of key design dimensions, Figure 3 operationalizes these insights into concrete, implementable design requirements across strategy, scope, structure, skeleton, and surface layers. This integration ensures that the framework not only defines what matters for refugee-centered design but also demonstrates how these requirements can be translated into actionable system design. The findings stress the importance of age-responsive and inclusive design, reflecting differences in preventive health priorities, information preferences, and interaction patterns among younger, middle-aged, and older women. These differences are not discrete but overlapping, requiring adaptable solutions rather than separate systems. In this regard, multimodal content formats support accessibility across abilities, literacy levels, and situational needs.

A central principle underpinning the framework is that trust and verification must be embedded by design. Trust operates across multiple dimensions, including the credibility of digital information, perceptions of healthcare institutions, and expectations shaped by prior experiences, and is operationalized within the framework through concrete design features. Trust is modeled by advertisement-free environments, transparent and verifiable information sources, professional translation, moderation of shared spaces, and visible alignment with public healthcare institutions. In this context, trust functions as a prerequisite for sustained engagement rather than an additional feature. Closely related is language and cultural adaptation, which extends beyond translation to include culturally meaningful communication. Providing Ukrainian as a default language, using trauma-sensitive wording, and incorporating culturally aligned visual representations can help bridge differences between prior healthcare experiences and the expectations of the host healthcare system.

The framework also stresses the importance of navigation and pathway guidance, where clear orientation, step-by-step support, and actionable pathways enable users to understand what to do, where to go, and when to act within the healthcare system. Age-related differences are incorporated through adaptable interaction pathways, multimodal content delivery, and flexible navigation structures, as detailed in Figure 3, enabling multiple user pathways within a unified system. Within this ecosystem, AI-supported features can play a complementary role in supporting orientation and information retrieval. However, their role should remain bounded and transparent, clearly signaling their non-clinical function and providing visible pathways to professional care. Human contact and professional reassurance remain essential, particularly when health concerns are perceived as serious or uncertain. Finally, the framework underscores the role of cognitive ease, accessibility, and universal design as foundational design principles. Structured content, predictable navigation, and supportive visual design reduce cognitive load and help prevent information overload, reinforcing usability and accessibility as safety-critical components of digital health systems rather than purely aesthetic considerations.

### 4.5 Limitations

This study has several limitations that should be considered when interpreting the findings. First, the study focused exclusively on URW, which, while appropriate given the research aims, limits the transferability of the findings to other refugee populations or to men. Second, participants were recruited within a specific national context, and the findings may reflect characteristics of the Norwegian healthcare system and its digital infrastructure that differ from those of other host countries. Third, digital health tool use was self-reported through a brief pre–focus group questionnaire and may therefore be subject to recall bias or social desirability bias. Fourth, the Ukrainian healthcare system prior to the conflict was highly digitalized, and so was its population. Consequently, the challenges identified in this study may not necessarily reflect limited digital literacy or insufficient digital engagement. Rather, they may be more strongly associated with language adaptation, differences between healthcare systems, and the integration of refugees into unfamiliar digital health ecosystems. Finally, this study focused on participants' perceived experiences and expectations rather than direct observation of digital system use. Future research could complement these findings through usability testing, longitudinal studies, or participatory co-design approaches involving both users and healthcare providers.

## 5 Conclusions

This study demonstrates that digital health engagement among URW is shaped primarily by their ability to navigate unfamiliar systems, access trustworthy information, and overcome language barriers. These factors emerged as key determinants of whether digital health information can be accessed, understood, and translated into action. The findings also highlight important age-related differences in digital health practices, with younger, middle-aged, and older women facing distinct challenges and priorities that need to be reflected in the design of equitable DHIs.

Building on these insights, a refugee-centered framework was developed to translate identified needs into design-relevant components. The framework emphasizes navigation and pathway guidance, age-responsive design, trust and verification, language and cultural adaptation, AI-supported features, and accessibility. Importantly, the findings should be interpreted in light of participants' prior experience with highly digitalized healthcare systems. This suggests that the barriers identified are less related to basic digital literacy and more to adapting to new institutional, linguistic, and technological environments.

The findings have clear implications for healthcare providers, policymakers, and digital health developers. Improving digital health access for refugee populations requires not only the provision of information but also systems that support orientation, build trust, and enable users to act confidently within unfamiliar healthcare environments. Addressing language barriers, ensuring trustworthy sources, and providing clear guidance are essential for supporting equitable healthcare access.

By grounding the framework in the lived experiences of URW, the study offers a practical foundation for designing DHIs that can better respond to their needs. Future research should validate and refine the framework in real-world settings and explore its applicability across different refugee populations and healthcare systems. Overall, the findings highlight the importance of user-centered design of digital health involving end users to ensure they are navigable, trustworthy, linguistically accessible, and responsive to the diverse needs, preferences, and experiences of different user groups.

## Data Availability

The raw data supporting the conclusions of this article will be made available by the authors, without undue reservation.
