# Supplementary material for: Refugee-centered framework for design of digital health interventions: a qualitative study with Ukrainian refugee women in Norway
Source: Front Digit Health. 2026 Jul 27;8:1886871. doi: 10.3389/fdgth.2026.1886871 (PMC13454123; doi:10.3389/fdgth.2026.1886871)
Supplement: Supplementary file 1 [file Supplementaryfile1.docx]

**Pre-Questionnaire for Ukrainian refugee women**

**Section 1: Background**

1. Your age: _____ years
2. Which place do you come from in Ukraine _____
3. How long have you lived in [country]? _____ months/years
4. Which of the following best describes your current primary living arrangement in the host country?
   (Please select one)
   - ☐ I am currently living alone (no children, partner, or other family members)
   - ☐ I am living with my partner/spouse only (no children or other family)
   - ☐ I am living with my minor children only (as a single parent household)
   - ☐ I am living with my partner/spouse and minor children
   - ☐ I am living with other family/relatives only (e.g., parents, siblings, or adult children, but no spouse/partner or minor children)
   - ☐ I am living with a host family or non-family members (e.g., roommates, in a shared asylum center)
   - ☐ Other: ______________________

**Section 3: Digital Tool Use and Trust**

1. **What electronic devices do you use frequently?**
   (Tick all that apply)
   - ☐ Smartphone/Mobile Phone
   - ☐ Laptop/Desktop Computer
   - ☐ Tablet (e.g., iPad)
   - ☐ None of the above
2. **Did you use a digital tool for accessing health information in your host country?**
   (Select one)
   - ☐ Never
   - ☐ Rarely
   - ☐ Sometimes
   - ☐ Often
3. **Can you please name the digital tool or app you used?**

**Focus Group Questions for Ukrainian refugee women**

**Section 4: Digital Health**

**Prior usage and preferences on Digital tool**

1. Can you describe your previous experiences with digital tools for health? Based on your experience with such tools- Imagine a website designed to help Ukrainian women with their health what type of information would be most useful for you? *Prompt: For example, would you prefer information about specific diseases, or information about how to make appointments and access healthcare services? What would be the most useful thing for it to have?*
2. What type of content do you prefer in health information? *Prompt: like personal stories from other URW, videos from doctors, or simple text and pictures—would make you feel the information is most trustworthy and easy to use? Why?*
3. What are the top one or two things that would encourage you to use a digital health tool regularly?
4. Conversely, what would make you stop using it immediately?
